# Maternal immunization impairs lymphoma growth and CNS/ocular metastasis in the offspring

**DOI:** 10.3389/fimmu.2024.1498272

**Published:** 2024-12-18

**Authors:** Ori Braitbard, Allan Bar-Sinai, Jacob Hochman

**Affiliations:** ^1^ Alexander Silberman Institute of Life Science, The Hebrew University of Jerusalem, Jerusalem, Israel; ^2^ Department of Bioinformatics, The Faculty of Life and Health Sciences, Jerusalem College of Technology, Jerusalem, Israel

**Keywords:** MMTV, MMTV p14, brain metastases, T-cell lymphoma, maternal immunization, CNS/ocular metastasis

## Abstract

Maternal immunization is an important tool directed against a variety of infectious maladies in the offspring. A complementary, but less explored area is the use of maternal immunization in the prevention and treatment of childhood cancers. This in part stems from the lack of adequate experimental model systems. Lymphomas of the Central Nervous System (CNS) and ocular involvement pose a therapeutic challenge. Ocular lymphoma is a lethal disease caused mainly by two clinically distinct forms of non-Hodgkin’s lymphoma: non-Hodgkin’s lymphoma of the central nervous system, or Primary CNS lymphoma (PCNSL), and systemic lymphoma metastatic to the eye. Previously, we developed an experimental model whereby mouse lymphoma cell variants, derived from the S49 T-cell lymphoma, metastasized to the CNS and eyes following Intraperitoneal inoculation at days 7-10 postnatal. Here, we extended the model to study whether maternal immunization can impede CNS/Ocular metastasis in the offspring exposed to the metastatic lymphoma cells. To that effect, female Balb/C mice were vaccinated with either immunogenic, live, S49 lymphoma cell variants, or with a purified protein antigen: the 98 amino acid signal peptide of the envelop precursor protein of Mouse Mammary Tumor Virus (MMTV) endogenously harbored by the S49 lymphoma. The offspring from both vaccination protocols were immunized against a challenge with the CNS/Ocular metastatic lymphoma cells. Immunity was conferred via milk suckling and was prolonged without further challenge for an extended period of at least 3 months. The abovementioned findings constitute a novel experimental model system whereby CNS/Ocular metastasis of malignant lymphoma in the offspring is impeded through maternal vaccination/immunization and thus, can be followed mechanistically as well as for novel therapeutic modalities.

## Introduction

The concept of maternal immunization protecting offspring from cancer represents a promising preventive strategy in oncology. Previous studies have demonstrated that maternal antibodies, transferred both through the placenta and breast milk, can provide crucial early-life immunity against various pathogens and diseases (1).

Cancer cell metastasis to the CNS is very often associated with a grim prognosis. Animal models have been critical in making progress toward understanding the pathogenesis of Ocular and CNS lymphomas and investigating potential therapeutic modalities (2–5).

Ocular lymphoma is a lethal disease caused mainly by two clinically distinct forms of non-Hodgkin’s lymphoma, non-Hodgkin’s lymphoma of the central nervous system, or Primary CNS lymphoma (PCNSL), and systemic lymphoma metastatic to the eye.

**Figure 1:**
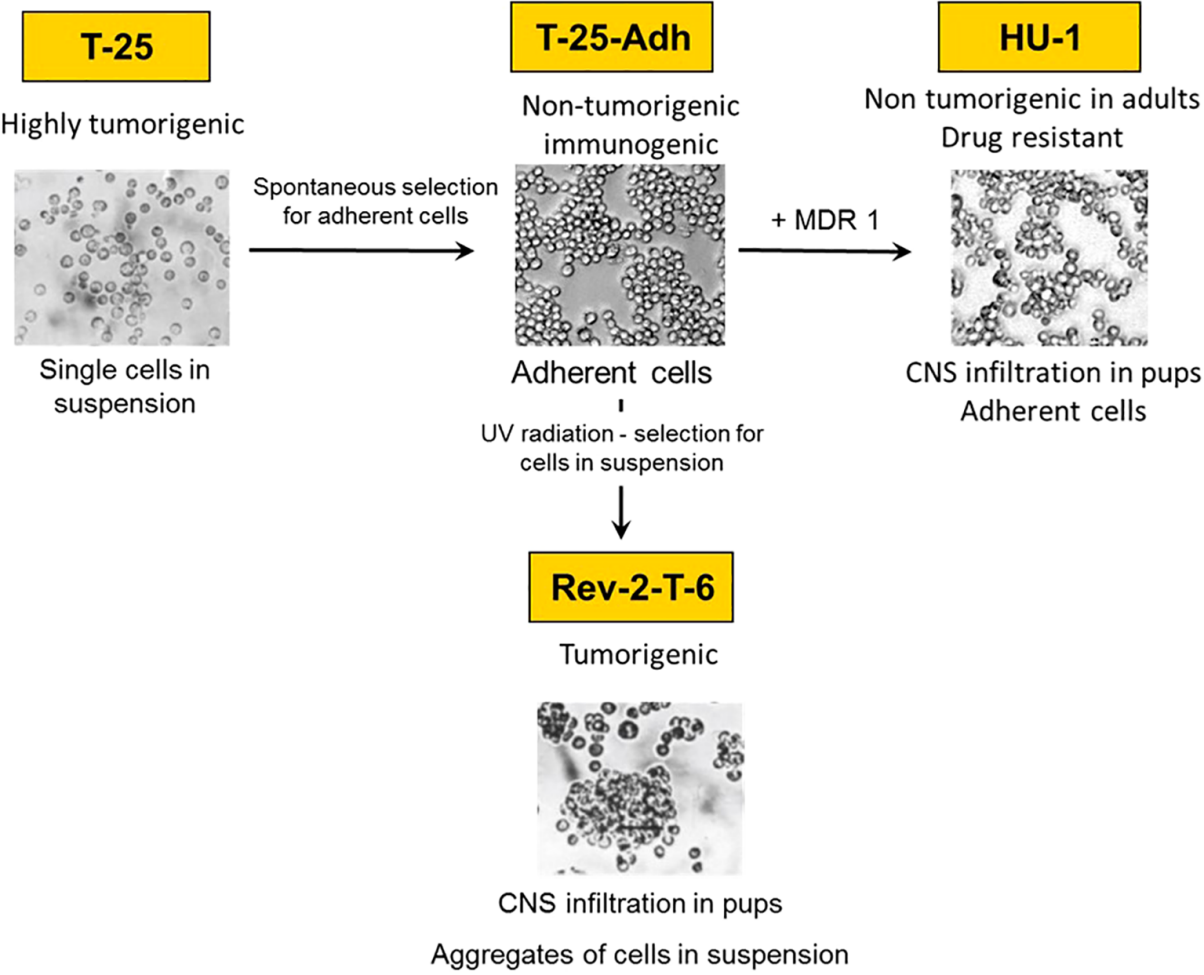
Lineage of the CNS lymphoma metastasis experimental model system.

Previously, we developed two experimental models for CNS/Ocular lymphoma metastasis: In one, Rev-2-T-6 cells [see below and **Figure 1**)] introduced intraperitoneally (IP) into syngeneic Balb/C mice at days 7-10 postnatal, metastasized to the brain and spread within it, reminiscent of CNS acute lymphoblastic lymphoma, Primary CNS, and Intraocular lymphoma. Infiltration of the brain occurred preferentially via the choroid plexus and cranial nerves (6–9). Within the brain, the lymphoma cells infiltrated the rostral part prior to the caudal part and moved along the fibers of the corpus callosum connecting the left and right cerebral hemispheres. In addition, the lymphoma cells migrated along the optic nerve sheath into the eyes, involving the vitreous, choroid, ciliary body, iris, culminating in the anterior chamber (6, 7) The eyelids and orbit were also infiltrated, albeit independent of the brain-optic-nerve-intraocular route (7). In a complementary, retrograde model, Rev-2-T-6 cells introduced into mature mice’s vitreous infiltrated the brain as well as the contralateral eye (10).

Rev-2-T-6 cells are one element of a unique experimental model system developed from the S49 murine T-cell lymphoma (11, 12) as follows: Parental S49 cells were subjected to continuous *in vivo* passages in syngeneic hosts. The resultant (highly tumorigenic) cells named T-25 were maintained as single cells in suspension culture and as ascites *in vivo* (median survival 17-20 days). From T-25 cells, we selected substrate adherent cell variants, named T-25-Adh, that grew as a monolayer (**Figure 1**). These cells were non-tumorigenic upon inoculation into immune-competent syngeneic hosts. Furthermore, intraperitoneal (IP) inoculation of live T-25-Adh cells into mature Balb/C mice immunized the recipients (whole cell vaccination) against a challenge with parental T-25 lymphoma cells.

Subsequently, from T-25-Adh cells, the abovementioned Rev-2-T-6 cells were derived. These, again, grow in suspension, albeit in clumps, and produce progressive, solid abdominal tumors with extended median survival (55-70 days) following IP inoculation into mature Balb/C mice. Thus, a full cycle was established from tumorigenic to non-tumorigenic (immunogenic) and back to a tumorigenic state, including CNS/Ocular metastasis (**Figure 1**).

Later, we observed that T-25-Adh cells, transduced with a retrovirus containing the human MDR1(ABCB1) cDNA (13), and rendered multi-drug resistant, also demonstrated CNS/Ocular metastasis, following inoculation into day seven post-natal mice. These cells, named HU-1grow in a monolayer and are non-tumorigenic in mature mice, similar to T-25-Adh cells (**Figure 1**) from which they were derived.

It is noteworthy that all cells of the S49 lineage endogenously harbor the Mouse Mammary Tumor Virus – MMTV. Previously, we demonstrated that the vaccinating potential of T-25-Adh cells is governed, at least in part, by the differential response of MMTV to interferon-mediated signaling pathway (14).

MMTV is a Beta-retrovirus that causes mammary carcinoma or lymphoma in mice. An increasing body of evidence in recent years supports the involvement of a virus that is genetically indistinguishable from MMTV in up to 38% of sporadic human breast cancer (15–17) (and references within). In that context, due to its electrophoretic mobility, we investigated the unusually long (98 amino acids) signal peptide of the 73kDa envelope precursor protein of MMTV (named MMTV-p14 or p14 for short). Its mass was subsequently determined by mass spectrometry as 11kDa (18) (**Supplementary Figure S1**). p14 localizes to nucleoli (19) and is also expressed on the surface of cells that harbor the virus (including the S49 cell lineage) (19, 20). Consequently, p14 was used both as a vaccine for tumors that contain the virus and for the generation of monoclonal antibodies used for immune therapy of murine lymphomas or mammary carcinomas that harbor the virus. Furthermore, antibodies to p14 have also been applied for the diagnosis of human breast cancer associated with the virus (18, 20–22).

Here, we extended the above model system to ask whether maternal vaccination could immunize the offspring against a challenge with CNS/Ocular metastatic lymphomas (Rev-2-T-6 and HU-1). To that effect, two approaches were tested:

1) – Maternal vaccination with live (immunogenic) T-25-Adh cells followed by offspring exposure to the metastatic Rev-2-T-6 cells.
2) – Maternal vaccination with a single peptide antigen, the above-mentioned p14, followed by exposure of the offspring to the metastatitc HU-1 cells (19, 20, 23).

## Materials and methods

### Cells

Cells were grown in a medium containing 88% RPMI-1640, 10% fetal bovine serum (FBS) or horse serum (HS), 1% L-glutamine, and 1% penicillin-streptomycin antibiotics (Biological Industries, Beit-Haemek, Israel), maintained under 5% CO_2_ at 37°C.

### Mice

Balb/C female mice were obtained from Harlan (Israel) and maintained in an SPF facility (AAALAC accreditation #1285). Mice were treated per NIH guidelines and approved by the Institutional Ethics Committee for Animal Studies (IACUC: No. NS-16-14848-5, NS-23-17168-5). For immunization, female Balb/C mice were vaccinated thrice intraperitoneally, at monthly intervals, with 5x10^6^ T-25-Adh cells or intramuscular with 1mg/mouse/injection p14 in Alum as adjuvant. Following pregnancy (1 month after the last cells or p14 inoculation), their offspring were challenged with 1x10^6^ Rev-2-T-6 or HU-1 cells at day seven post-natal and followed thereafter for clinical signs of Ocular and CNS involvement.

### Milk collection

Nursing female mice separated from their offspring for 5 hours were injected IP with 8-10 units of Oxytocin. Micropipette suction was used for milk collection and stored at -20°C until use.

Rev-2-T-6 produces progressive, solid abdominal tumors following intraperitoneal inoculation into mature Balb/C mice. These cells demonstrate cell–cell adhesion, growing in suspension culture as cell aggregates. HU-1 cells derived from T-25-Adh cells express the ABCB1 transporter (the human MDR-1) (see text for details).

### Inoculation of pups

Rev-2-T-6 and HU-1 cells (1 × 10^6^) were inoculated IP into syngeneic Balb/C mice on day 7 postnatal. Mice were checked daily for palpable abdominal tumors, as well as for signs of eye and CNS involvement.

### Western blots

Cells were lysed by SDS-PAGE sample buffer, separated by 15% SDS-PAGE, and transferred to nitrocellulose. Protein extracts were visualized using the polyclonal serum or milk from vaccinated females described above, HRP-linked donkey anti-mouse antibody (Jackson Laboratories), and Pierce Super Signal.

### Statistical analyses

Two-way ANOVA with Bonferroni multiple comparison post-test was operated for survival over time utilizing GraphPad Prism 5.04 software. Statistical significance was defined at the level of P < 0.05.

Statistical analysis was also performed using the log-rank (Mantel-Cox) test to compare survival (Kaplan-Meier) distributions between the two experimental groups. Survival curves were generated using GraphPad Prism (version 5.04, GraphPad Software, San Diego, CA). Statistical significance was set at p<0.05.

## Results

### Maternal vaccination with live cells

The offspring of Balb/C females inoculated IP with live T-25-Adh cells were subjected to an IP challenge with Rev-2-T-6 cells at day seven post-natal.

**Figure 2:**
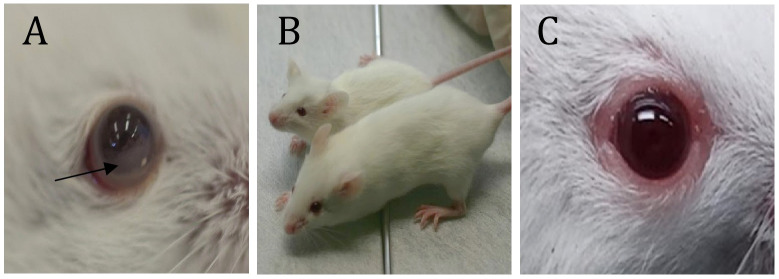
Some visible symptoms of CNS and Ocular metastasis by lymphoma. **(A)** - Hypopyon-like the manifestation of lymphoma cells in the anterior chamber; **(B)** – Growth retardation (compared to naïve mice of the same age); **(C)** – Eyelids and Orbital involvement. *(D) - Twist when held by the tail. *; see **Supplementary Video** (**Supplementary Figure S2**).

**Figure 3:**
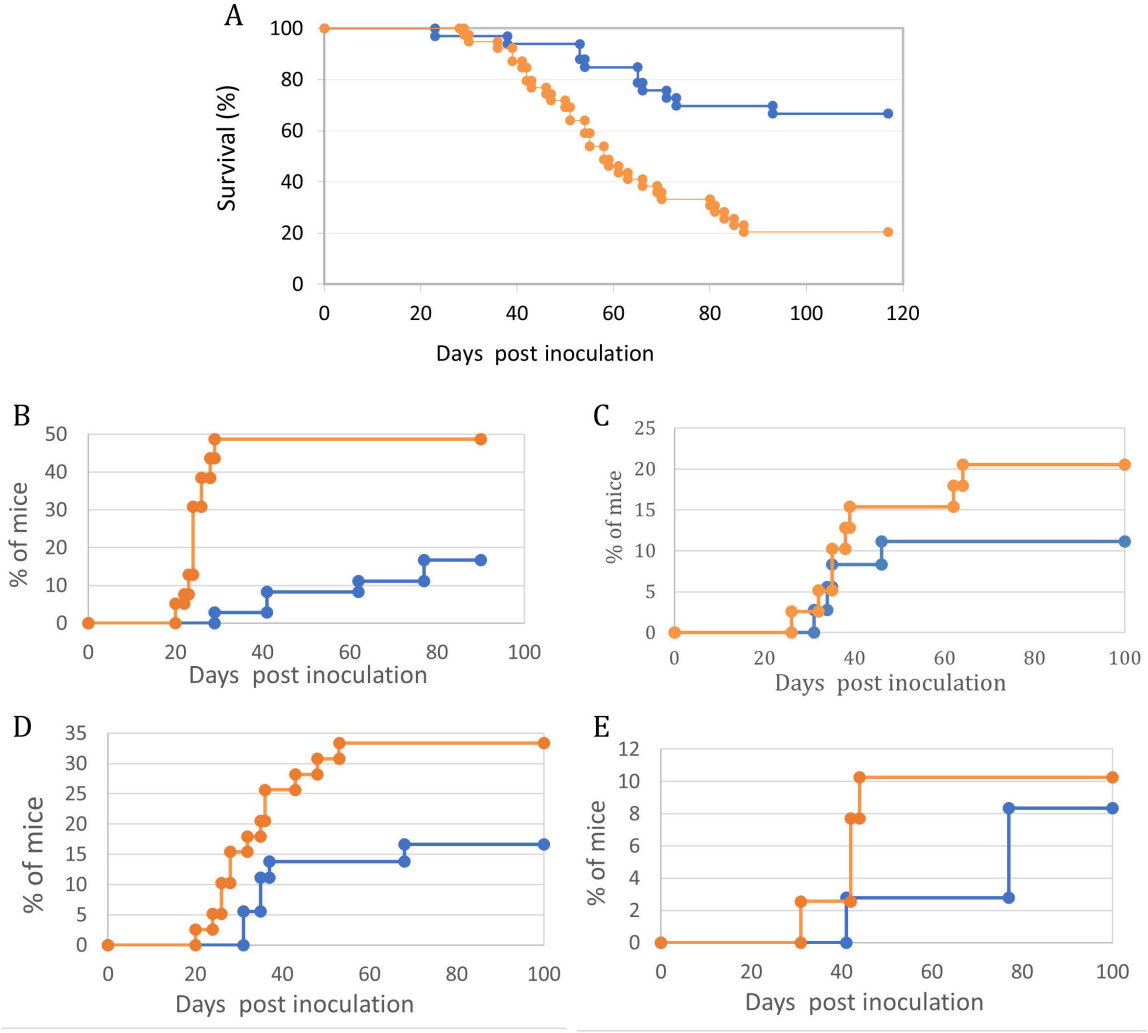
Maternal vaccination with T-25-Adh cells enhances survival and decreases symptoms of lymphoma growth and Ocular/CNS metastasis in their offspring challenged with Rev-2-T-6 cells at day 7 post-natal. In all graphs, Orange = Control, no maternal vaccination (n=39); Blue- Experimental, maternal vaccination (n=36). **(A)** – Survival. **(B)** – Abdominal tumor masses. **(C)** – CNS involvement. **(D)** – Eyelids and Orbit. **(E)** – Anterior chamber. Kaplan-Meier survival curves- **(A)** showing differential survival patterns between treatment (blue line) and control (orange line) groups over 120 days post-inoculation. The treatment group maintained significantly higher survival (p < 0.001, log-rank test), with 68% of subjects surviving to study completion. The control group showed progressive mortality beginning at day 30, with median survival of 58 days and final survival rate of 20%. The divergence in survival curves became apparent by day 40 and continued throughout the observation period, indicating robust and sustained protection in the treatment group. Initial survival was comparable between groups until day 30, suggesting that treatment primarily affects disease progression rather than early pathogenic events. Four distinct **(B–E)** manifestation patterns were monitored in treated (blue line) and control (orange line) groups (n = 30-35 mice per group). Statistical analysis revealed significant differences between groups for all parameters (p < 0.001 for all comparisons). The control group showed consistently higher disease progression rates, with particularly marked differences emerging between days 20-40 post-inoculation. Peak manifestation rates were reached by day 60 in most parameters, with the control group showing plateaus at higher levels (33%, 10%, 48%, and 20% for parameters **(B–E)** respectively) compared to the treatment group (17%, 8%, 17%, and 11%). Notable stepwise progression was observed in Abdominal tumor masses -**(B)**, suggesting distinct phases of disease development. Disease onset occurred earlier in the control group across all parameters, with the most pronounced temporal difference observed in CNS involvement -**(C)**. Data points represent mean percentages of affected mice at each time point throughout the 100-day observation period.

These were followed for systemic, CNS, and Ocular involvement symptoms. (**Figure 2**). It is evident that by all parameters tested, ie. Overall survival (**Figure 3A**), Abdominal palpable tumor masses, CNS associated signs, Anterior chamber (Hypopion like), and Orbital involvement (**Figures 3B–E**), the offspring from vaccinated mothers fared better than the offspring of none vaccinated mothers (65% *vs*. 20% survival, respectively) as well as delayed onset of the above-mentioned visible symptoms.

CNS and Ocular involvement were monitored through clinical signs such as hind leg paralysis, growth inhibition, twist, and finally the appearance of cells in the anterior chamber of the eye. In our previously published experiments, we demonstrated the migration pathway of lymphoma cells within the brain using detailed (serial sections) histopathological analysis.

The association between the histopathological analyses and the clinical signs of CNS and Ocular involvement were already established in our earlier publications on the topic (6, 7).

**Figure 4:**
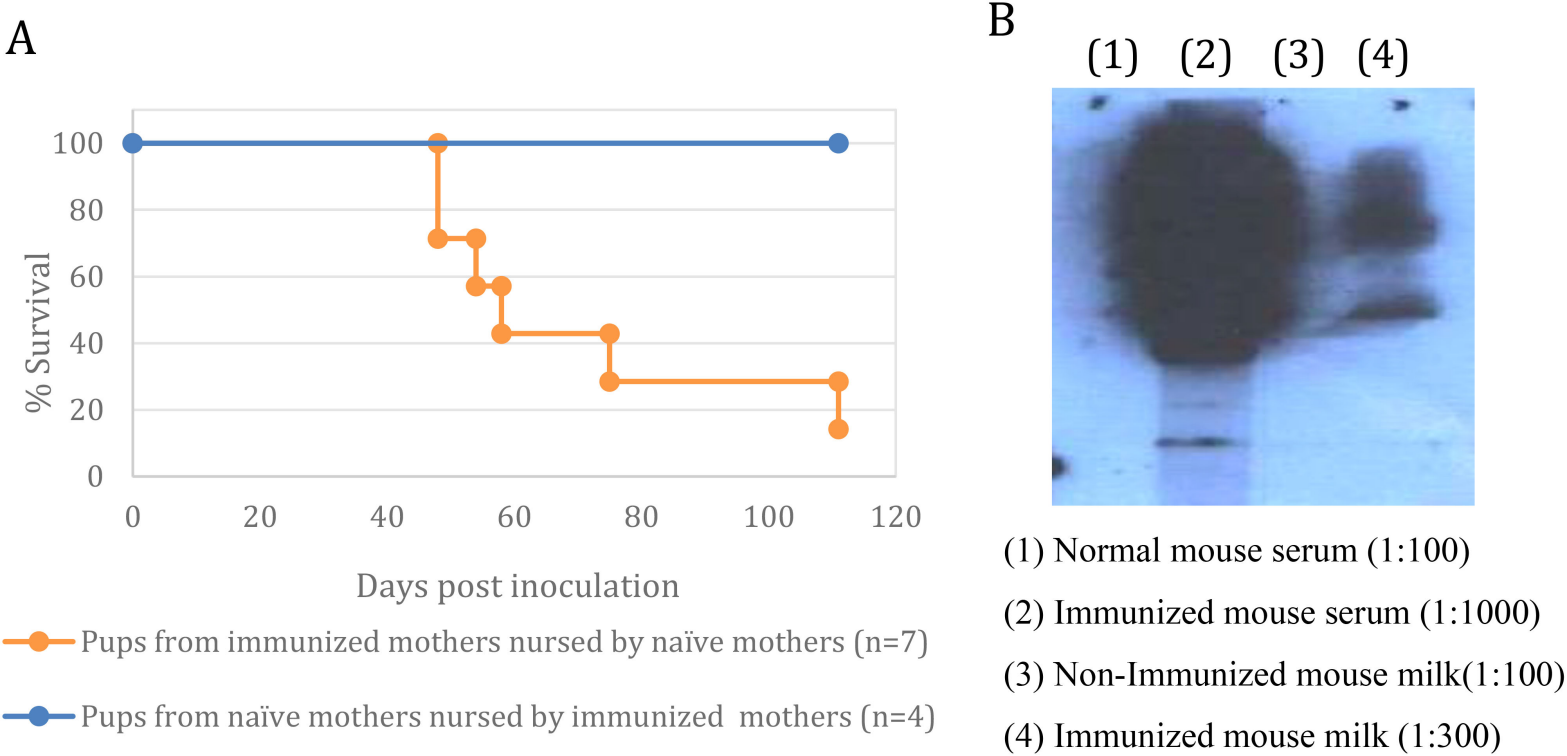
Maternal immunization is transferred to offspring via nursing. **(A)** – Exchange of mothers - The progeny of mothers immunized with T-25-Adh cells were exchanged with the offspring of non-immunized mothers within 24 hours postpartum. Both groups were challenged with Rev-2-T-6 cells at day 7 postnatal and followed thereafter. Median survival: control 60 days, experimental <120 days (*** P ≤ 0.001 relative to naïve group). Survival rates of mouse pups under cross-fostering conditions demonstrates maternal antibody transfer via nursing. Pups born to immunized mothers but nursed by naïve mothers (orange line, n=7) showed progressive mortality starting at day 45 post-inoculation, reaching 15% survival by day 120. In contrast, pups from naïve mothers nursed by immunized mothers (blue line, n=4) maintained 100% survival throughout the observation period, suggesting that protective antibodies are effectively transferred through lactation rather than *in utero*. Survival curves were compared using log-rank test. **(B)** – Anti-T-25-Adh antibodies in milk and serum of immunized *vs*. non-immunized females.

Exchanging the offspring between immunized and naïve mothers during day one postnatal, followed by challenging both groups with Rev-2-T-6 cells, demonstrated that immunity towards the lymphoma cells is transferred from mother to offspring via nursing (**Figure 4A**). It is evident that in this model system, transplacental transfer of anti-lymphoma antibodies from immunized mothers to offspring does not play any significant role postpartum.

Western blot analysis demonstrated that serum and milk from immunized females contained anti–T–25–Adh antibodies. In contrast, serum and milk from naïve females were devoid of such antibodies (**Figure 4B**). The concentration of antibodies in the serum of immunized females is at least 30-fold higher than in their milk.

The response to immunization using whole live T-25-Adh cells or purified p14 was both humoral (IgG) as well as cellular (Adoptive T-cell transfer from immunized to naïve mice) (6, 12, 20). A battery of monoclonal antibodies directed against p14 and derived from immunized mice were all the IgG type.

**Figure 5:**
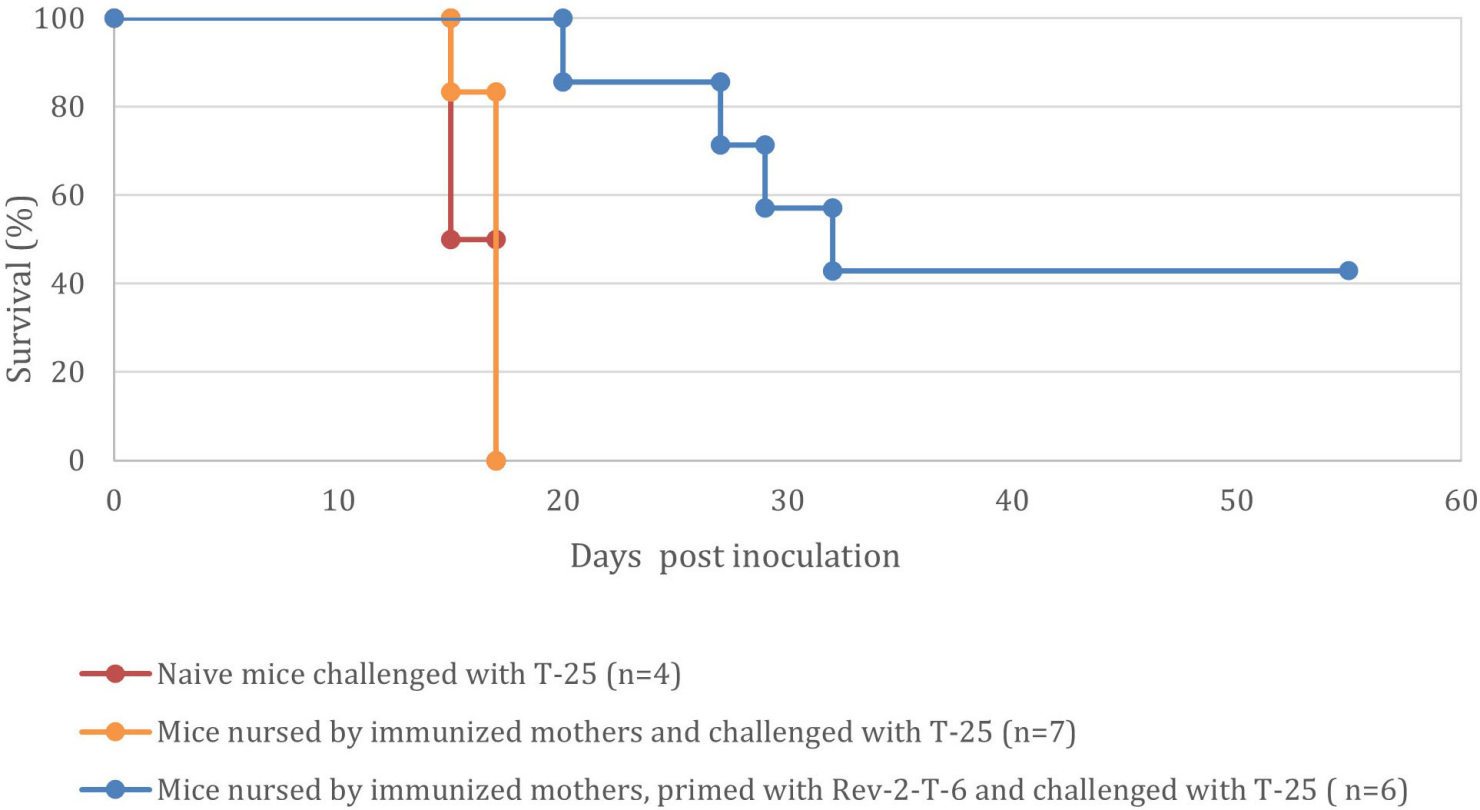
Mice nursed by immunized mothers and withstood the challenge with Rev-2-T-6 cells demonstrate prolonged resistance to a subsequent challenge (at 90 days post inoculation) with the parental, highly tumorigenic, T-25 cells (P < 0.001 relative to the naive group). Kaplan-Meier survival curves showing the percentage survival of naive mice (red line, n=4), mice nursed by immunized mothers (orange line, n=7), and mice nursed by immunized mothers and primed with Rev-2-T-6 (blue line, n=6) following challenge with T-25. Median survival times were 17 days for naive mice, 17 days for nursed mice, and 30 days for nursed and primed mice. While nursing alone did not significantly improve survival compared to naive mice (p = 0.92), the combination of maternal nursing and priming significantly extended survival compared to both naive and nursed-only groups (p < 0.001 for both comparisons). The primed group maintained 42% survival at study completion (60 days), demonstrating the synergistic protective effect of maternal antibodies and active immunization. Data represents pooled results from two independent experiments.

Mice that were nursed by immunized mothers and withstood the challenge with Rev-2-T-6 cells demonstrated enhanced survival when challenged three months later with the highly tumorigenic parental T-25 cells (**Figure 5**).

### Maternal vaccination with a single peptide antigen

To determine whether a single antigen vaccination is sufficient to transfer immunity from mother to offspring, mice were subjected twice to intramuscular (IM) injection of recombinant p14 (20) and the offspring were subjected to an IP challenge with HU-1 lymphoma cells at day seven post-natal.

**Figure 6:**
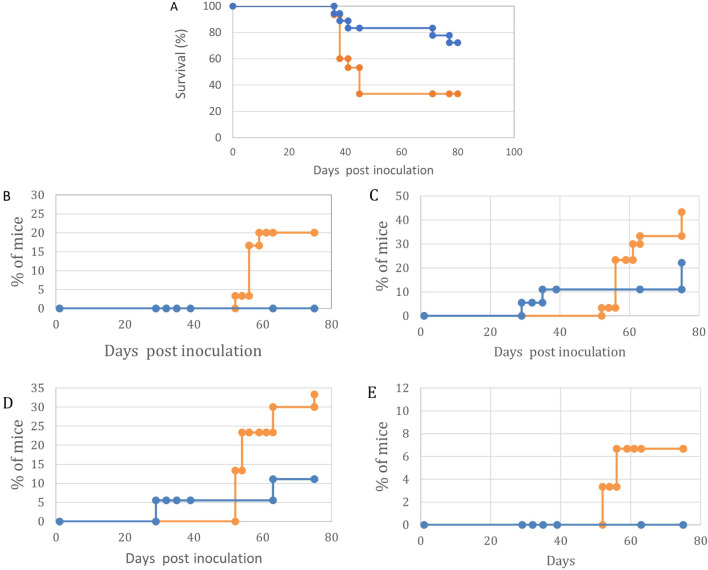
Maternal vaccination with p14 enhances survival and impairs symptoms of lymphoma growth and Ocular/CNS metastasis in their offspring challenged with HU-1 cells at day 7 post-natal. In all graphs, Orange – Control, no maternal vaccination (n=30); Blue- Experimental, Maternal vaccination (n=17). **(A)** – Survival. **(B)**- Abdominal tumor masses. **(C)**- CNS involvement **(D)** – Eyelids and Orbit. **(E)**- Anterior chamber. Kaplan-Meier survival curves demonstrate differential survival patterns between treatment (blue line) and control (orange line) groups over 80 days post-inoculation. The treatment group exhibited significantly improved survival (p < 0.001, log-rank test) with 72% of subjects surviving at day 80 and median survival not reached. In contrast, the control group showed rapid decline in survival between days 40-48, with median survival of 45 days and final survival rate of 35%. Initial mortality events occurred simultaneously in both groups around day 40, suggesting a critical threshold in disease progression, but subsequent survival trajectories diverged significantly. These results indicate substantial protective effects of the treatment regimen, particularly in preventing late-stage mortality. Following tumor cell inoculation, mice were monitored for 80 days (Control n=30, Experimental n=17). The experimental group demonstrated significantly reduced metastatic burden in orbit, abdominal cavity, and anterior chamber (all p<0.01), with 50% lower incidence compared to controls at day 80. Overall survival was significantly improved in the experimental group (p<0.001). The four panels **(B-E)** depict the temporal patterns of disease manifestation in control (orange) and experimental (blue) groups following inoculation, including orbital inflammation **(B)**, abdominal tumor development **(C)**, anterior chamber involvement **(D)**, and neurological signs **(E)**. Statistical analysis revealed significant differences between groups for orbital inflammation (p < 0.001), abdominal tumors (p < 0.01), and neurological signs (p < 0.001), while anterior chamber involvement showed no significant difference (p = 0.089). The experimental group consistently exhibited lower rates of disease progression across the majority of parameters, with the most pronounced effects observed for orbital and neurological manifestations. Notably, the control group exhibited rapid, stepwise increases in disease incidence for orbital, abdominal, and neurological endpoints, suggesting critical thresholds in pathogenesis. In contrast, the experimental group demonstrated more gradual, attenuated progression of these complications. These results demonstrate the potent protective effects of the experimental intervention in mitigating multiple facets of the disease process.

It is evident (**Figure 6**) that maternal vaccination with p14 impedes tumor development and enhances survival in offspring challenged with lymphoma cells, similar to the findings with whole cell vaccination (**Figure 3**). Visible symptoms are shown in **Figure 2**.

**Figure 7:**
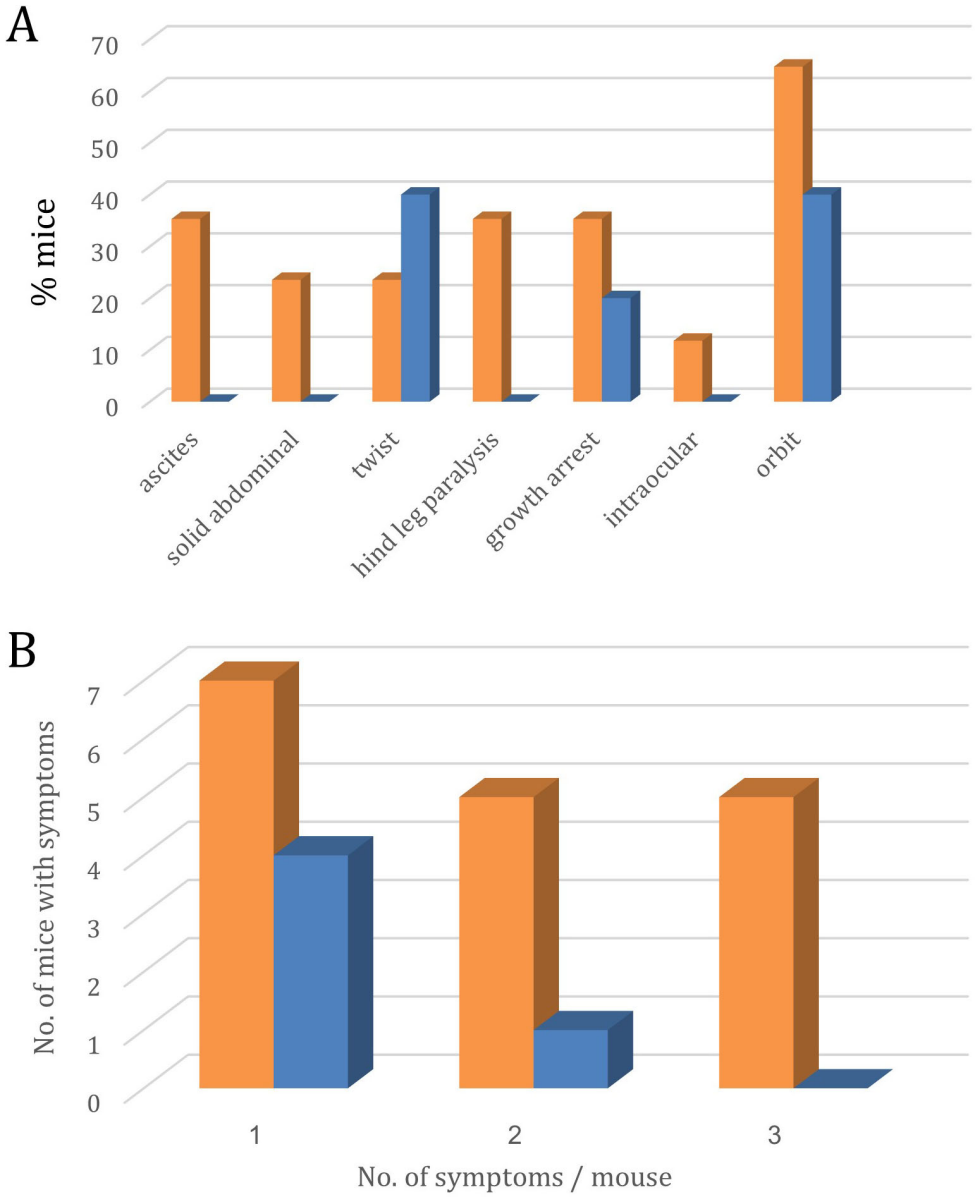
Symptoms of lymphoma metastasis to the eye and CNS in the offspring of p14-immunized (Blue, n=18) and non-immunized (Orange, n=30) mothers. **(A)** – Site distribution of symptoms.; **(B)** – No. of mice expressing 1, 2, or 3 different symptoms.

Two additional criteria that demonstrate the efficacy of single antigen maternal vaccination are 1) The increased number of visible clinical signs in non-immunized pups *vs*. immunized pups (**Figure 7A**) and the number of different clinical signs per pup (**Figure 7B**).

Bar graph A shows the percentage of mice exhibiting various pathological features under control (orange) and experimental (blue) conditions. Significant differences were observed in twist presentation (40% experimental *vs* 23% control, p<0.05), growth arrest (20% experimental *vs* 35% control, p<0.05), and orbital involvement (40% experimental *vs* 65% control, p<0.01). Additional manifestations observed exclusively in control animals included ascites (35%), solid abdominal masses (23%), hind leg paralysis (35%), and intraocular involvement (12%). These results suggest the experimental intervention significantly modifies disease progression and reduces overall symptom burden.

Bar graph B depicts the average number of symptoms observed per mouse in the control (orange) and experimental (blue) groups, categorized by the number of symptoms (1, 2, or 3).

Statistical analysis using the Kruskal-Wallis test revealed a significant difference in symptom burden between the two groups (p = 0.0134). Mice in the control group exhibited a higher average number of symptoms across all categories compared to the experimental group.

Notably, for mice experiencing 3 symptoms, the control group had an average of 6.0 mice per observation, whereas the experimental group had 3.25 mice per observation. This suggests the experimental intervention was effective in reducing the severity and prevalence of the most advanced disease manifestations.

The data demonstrate the beneficial impact of the experimental treatment in mitigating the overall symptom burden experienced by the mice, with the most pronounced effects seen in the highest symptom category.

By the parameters tested (**Figures 7A, B**), the offspring of immunized females had a better chance of protection from brain and ocular involvement symptoms, including extended survival.

## Discussion

As shown in our experimental data, offspring from vaccinated mothers exhibited significantly reduced cancer symptom burden, with fewer mice developing multiple symptoms compared to the control group (p<0.001). This protective effect aligns with emerging evidence that maternal vaccination can modulate offspring immune responses beyond infectious diseases, potentially offering a novel approach to cancer prevention from the earliest stages of life. The mechanism likely involves the transfer of specific antibodies and immune factors that help prime the offspring’s immune system to better recognize and eliminate malignant cells.

The bulk of maternal immunization studies involves bacterial and viral infectious agents. A promising area of maternal immunization with, still, a very low number of experimental models available, is the prevention and treatment of cancer. Barutello G, et al. reported that maternal anti-tumor immunization delayed mammary tumor development in cancer-prone offspring (24). A recent review (25) addressed briefly this issue suggesting that childhood cancers like lymphoma and neuroblastoma may be most susceptible to maternal immunization. Here we demonstrate that maternal vaccination can be applied towards lymphoma that metastasizes to the brain and eyes.

Xenogenization is a term coined earlier by Hiroshi Kobayashi to describe all attempts at rendering tumor cells antigenically foreign to their host such that they might be used more effectively in immune therapy. We demonstrate here that maternal vaccination, either with whole, live, adherent (xenogenized) lymphoma (T-25-Adh) cells or with a single peptide (p14) antigen, immunizes the offspring against a challenge with lymphoma cell variants that metastasize to the brain and eyes. This immunity is transmitted through maternal nursing during the first 21 days postpartum when pups are separated from their mothers.

It is evident that in this model system, transplacental transfer of anti-lymphoma antibodies from immunized mothers to offspring occurs prenatally but does not play a significant role, as the above exchange of pups between immunized and non-immunized mothers demonstrated.

Mice nursed by vaccinated mothers and withstood the challenge with Rev-2-T-6 cells demonstrated enhanced resistance to a later (at day 90) challenge with the highly tumorigenic parental T-25 cells. Thus, while subjected to passive immunization through mother’s milk, concomitantly, the metastatic (Rev-2-T-6) cell variants induce an active, long-term immune response against T-25 lymphoma cells. These findings suggest that maternal vaccination can exert a longer lasting anti-lymphoma effect.

Whole (live) cell cancer vaccines are meant to induce an immune response against multiple cellular antigens, and at the same time also activate (if possible) intracellular signaling pathways that enhance the immunogenic potential of the xenogenized tumor cells. The interferon effect on T-25-Adh cells is an example for such a mechanism (14), namely, presence of interferon increases the immunogenic potential of T-25-Adh cells while the same cells rendered interferon resistant demonstrated impaired immunogenicity. Such pathways are unavailable in the case of fixed cells or cellular extracts. A manuscript by Cicchelero et al. reviewed various ways to improve whole cancer cell vaccines (26).

Live cell vaccination against cancer has limitations, ie: it could induce an autoimmune response that cross-reacts with other autologous, non-cancerous cells. This can be detrimental clinically.

Interestingly, while HU-1 cells (like T-25-Adh from which they were derived) are adherent and non-tumorigenic in mature mice, in contrast to T-25-Adh cells, they are metastatic to the eyes and brain, similar to Rev-2-T-6 cells. Both T-25 and T-25-Adh cells are essentially non-metastatic to the eyes and brain in young mice.

The results obtained, both by vaccination with whole (live) cells or with a single antigen, clearly demonstrated the ability to significantly reduce the percentage of tumor cells reaching the brain and eyes and indicate that our strategy can be a first step in developing treatments for tumors that infiltrate the brain and eyes.

The experimental model presented in the article can also be used to develop new types of chemotherapy, such as titanium compounds-based chemotherapy, we have been researching in recent years (27, 28).

The present model system is amenable to a variety of studies, including comparative genomic/proteomic and signaling analyses of the different cell line variants (metastatic *vs*. non-metastatic, tumorigenic *vs*. immunogenic, adherent *vs*. suspension borne, and cells in culture *vs* the same cells taken from the anterior chamber) thus defining molecular and immunological signaling pathways that take part in these processes.

In addition, this model can be used for developing a variety of therapeutic modalities

Attractive experimental models whereby brain metastasis by lymphoma cells can be studied are Zebra fish models of pediatric brain tumors (29) where the labelled lymphoma cells can be followed visually for brain infiltration.

The manuscript is a proof of concept study that models (in mice) how maternal immunization can help the nursed offspring resist CNS and ocular lymphoma.

## Data Availability

The raw data supporting the conclusions of this article will be made available by the authors, without undue reservation.

## Supplementary material

Supplementary Figure 1 p14 is the 98 amino acid signal peptide of the 73 kDa envelope precursor protein of the Mouse Mammary Tumor Virus.

## References

[1] Palmeira P Quinello C Silveira-Lessa AL Zago CA Carneiro-Sampaio M. IgG placental transfer in healthy and pathological pregnancies. Clin Dev Immunol. (2012) 2012:1–13. doi: 10.1155/2012/985646 PMC3251916 22235228

[2] Sagoo M Mehta H Swampillai AJ Cohen VML Amin SZ Plowman PN. Primary intraocular lymphoma. Survey Ophthalmol. (2014) 59:503–16. doi: 10.1016/j.survophthal.2013.12.001 24560125

[3] Nussenblatt B Chan CC Wilson WH Hochman J Gottesman M CNS Ocular Lymphoma Workshop Group. CNS and Ocular Lymphoma Workshop Group. International Central Nervous System and Ocular Lymphoma Workshop: recommendations for the future. Ocul Immunol Inflammation. (2006) 14:139–44. doi: 10.1080/09273940600630170 PMC2518223 16827214

[4] Jager MJ Cao J Yang H Decaudin D Kalirai H van der Ent W. Animal models of ocular tumors. In: Chan CC, editor. Animal Models of Ophthalmic Diseases Essentials in Ophthalmology. Springer, Cham (2015). p. 127–40.

[5] Aronow ME Shen D Hochman J Chan C. Intraocular lymphoma models. Ocular Oncol Pathol. (2015) 1:214–22. doi: 10.1159/000370158 PMC4847663 27171354

[6] Assaf N Hasson T Hoch-Marchaim H Pe’er J Gnessin H Deckert-Schlüter M. An experimental model for infiltration of Malignant lymphoma to the eye and brain. Virchows Archiv. (1997) 431:459–67. doi: 10.1007/s004280050124 9428935

[7] Hochman J Assaf N Deckert-Schlüter M Wiestler OD Pe’er J. Entry routes of Malignant lymphoma into the brain and eyes in a mouse model. Cancer Res. (2001) 61:5242–7. 11431365

[8] Chan C Fischette M Shen D Mahesh SP Nussenblatt RB Hochman J. Murine model of primary intraocular lymphoma. Invest Ophthalmol Vis Sci. (2005) 46:415–9. doi: 10.1167/iovs.04-0869 PMC1950335 15671263

[9] Frishman-Levy L Shemesh A Frenkel S Bar-Sinai A Ni Z Kaufman DS. The regulation of central nervous system acute lymphoblastic leukemia by natural killer cells. Blood. (2013) 122:1393–3. doi: 10.1182/blood.V122.21.1393.1393 PMC7265786 25896649

[10] Hochman J Shen D Gottesman MM Chan CC. Anti-LFA-1 antibodies enhance metastasis of ocular lymphoma to the brain and contralateral eye. Clin Exp Metastasis. (2013) 30:91–102. doi: 10.1007/s10585-012-9512-2 22865235 PMC3529209

[11] Hochman J Hochman J Katz A Levy E Eshel S. Substrate-adhering lymphoid cells show impaired tumorigenicity and increased immunogenicity. Nature. (1981) 290:248–9. doi: 10.1038/290248a0 7207615

[12] Hochman J Levy E Mador N Gottesman MM Shearer GM Okon E. Cell adhesiveness is related to tumorigenicity in Malignant lymphoid cells. J Cell Biol. (1984) 99:1282–8. doi: 10.1083/jcb.99.4.1282 PMC2113301 6480692

[13] Galski H Lazarovici P Gottesman MM Murakata C Matsuda Y Hochman J. KT-5720 reverses multidrug resistance in variant S49 mouse lymphoma cells transduced with the human MDR1 cDNA and in human multidrug-resistant carcinoma cells. Eur J Cancer. (1995) 31A:380–8. doi: 10.1016/0959-8049(94)00511-3 7786606

[14] Mador N Falk H Bergel M Panet A Hochman J. Variant mouse lymphoma cells with modified response to interferon demonstrate enhanced immunogenicity. Cancer Immunol Immunother. (1997) 44:249–56. doi: 10.1007/s002620050380 9247559 PMC11037654

[15] Bevilacqua G. The viral origin of human breast cancer: from the mouse mammary tumor virus (MMTV) to the human betaretrovirus (HBRV). Bevilacqua Generoso. (2022) 14:1704. doi: 10.3390/v14081704 PMC9412291 36016325

[16] Gunzburg WH Salmons B. The role of a betaretrovirus in human breast cancer: enveloping a conundrum. Viruses Vols. (2022) 14:2342. doi: 10.3390/v14112342 PMC9695795 36366440

[17] Fekete Z Tertan BO Raduly L Eniu DT Buiga R Galatar M. Prevalence of MMTV-like sequences in breast cancer samples in Romanian patients-there is a geographic difference compared to the Western world. Infect Agents Cancer. (2023) 18. doi: 10.1186/s13027-023-00486-y PMC10283304 37340312

[18] Bar-Sinai A Bassa N Fischette MR Gottesman MM Love DC Hanover JA. Mouse mammary tumor virus env–derived peptide associates with nucleolar targets in lymphoma, mammary carcinoma, and human breast cancer. Cancer Res. (2005) 65:7223–30. doi: 10.1158/0008-5472.CAN-04-3879 16103073

[19] Feldman D Roniger M Bar-Sinai A Braitbard O Natan C Love DC. The signal peptide of mouse mammary tumor virus-env: a phosphoprotein tumor modulator. Mol Cancer Res. (2012) 10:1077–86. doi: 10.1158/1541-7786.MCR-11-0581 22740636

[20] Braitbard O Roniger M Bar-Sinai A Rajchman D Gross T Abramovitch H. A new immunization and treatment strategy for mouse mammary tumor virus (MMTV) associated cancers. Oncotarget. (2016) 7:21168–80. doi: 10.18632/oncotarget.v7i16 PMC5008276 26934560

[21] Lawson JS Mazzanti CM Civita P Menicagli M Ngan CC Whitaker NJ. Association of mouse mammary tumor virus with human breast cancer: histology, immunohistochemistry and polymerase chain reaction analyses. Front Oncol. (2018) 8. doi: 10.3389/fonc.2018.00141 PMC5950654 29868468

[22] Naccarato AG Lessi F Zavaglia K Scatena C Hamad MA Aretini P. Mouse mammary tumor virus (MMTV) - like exogenous sequences are associated with sporadic but not hereditary human breast carcinoma(2019). Available online at: https://ncbi.nlm.nih.gov/pubmed/31518337. 10.18632/aging.102252 PMC6756874 31518337

[23] Hochman J Braitbard O. Life after cleavage: the story of a β-retroviral (MMTV) signal peptide-from murine lymphoma to human breast cancer. Viruses. (2022) 14:2435. doi: 10.3390/v14112435 36366533 PMC9694287

[24] Barutello G Curcio C Spadaro M Arigoni M Trovato R Bolli E. Antitumor immunization of mothers delays tumor development in cancer-prone offspring. OncoImmunology. (2015) 4. doi: 10.1080/2162402X.2015.1005500 PMC4485839 26155401

[25] Engmann C Fleming JA Khan S Innis BL Smith JM Hombach J. Closer and closer? Maternal immunization: current promise, future. J Perinatol. (2020) 40:844–57. doi: 10.1038/s41372-020-0668-3 PMC7223555 32341454

[26] . Cicchelero L de Rooster H Sanders NN. Various ways to improve whole cancer cell vaccines. Expert Rev Vaccines. (2014) 13:721–35. doi: 10.1586/14760584.2014.911093 24758597

[27] Miller M Braitbard O Hochman J Tshuva EY. Insights into molecular mechanism of action of salan titanium(IV) complex with *in vitro* and *in vivo* anticancer activity. J Inorg Biochem. (2016) 163:250–7. doi: 10.1016/j.jinorgbio.2016.04.007 27090292

[28] Meker S Braitbard O Hall MD Hochman J Tshuva EY. Specific design of titanium(IV) phenolato chelates yields stable and accessible, effective and selective anticancer agents. Chemistry. (2016) 22:9986–95. doi: 10.1002/chem.201601389 27320784

[29] Basheer F Dhar P Samarasinghe RM. Zebrafish models of paediatric brain tumours. Int J Mol Sci. (2022) 23:9920. doi: 10.3390/ijms23179920 36077320 PMC9456103

