# Supplementary figures and images for: Maternal immunization impairs lymphoma growth and CNS/ocular metastasis in the offspring
Source: Front Immunol. 2024 Dec 18;15:1498272. doi: 10.3389/fimmu.2024.1498272 (PMC11688474; doi:10.3389/fimmu.2024.1498272)

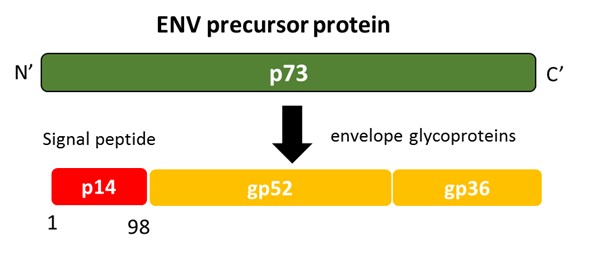

Supplement: Supplementary Figure 1 — p14 is the 98 amino acid signal peptide of the 73 kDa envelope precursor protein of the Mouse Mammary Tumor Virus. [file Image1.jpeg]
